# CHIP/STUB1 suppresses colorectal tumorigenesis through ubiquitin-mediated degradation of LDHA and inhibition of glycolysis

**DOI:** 10.1007/s13402-026-01260-z

**Published:** 2026-07-07

**Authors:** Chunya Li, Yanshen Kuang, Xinwen Hou, Linpeng Zhao, Yutong Zhang, Shaochang Wang, Peijia Wang, Zifan Dai, Xiaoxuan Wang, Yu Shang

**Affiliations:** 1 Key Laboratory of Cell Proliferation and Regulation of Ministry of Education, College of Life Sciences, Beijing Normal University, Beijing, 100875 China; 2 Department of General Surgery, Chinese PLA General Hospital, Beijing, 100141 China; 3 School of Life Science, Shanxi Normal University, Taiyuan, 030031 China; 4 Peking Academy High School, Beijing, 100009 China; 5 Molecular Diagnostics Center, Peking University People’s Hospital, Beijing, 100044 China

**Keywords:** CHIP, LDHA, Colorectal cancer, Glycolysis, Ubiquitination, H3K18la

## Abstract

**Background:**

The E3 ubiquitin ligase CHIP/STUB1 is a known tumor suppressor in colorectal cancer (CRC) and other malignancies, but its mechanistic role in metabolic reprogramming remains elusive. This study aimed to elucidate whether CHIP regulates glycolysis in CRC through targeting key metabolic enzymes.

**Methods:**

Lentiviral systems were employed to generate SW480 and DLD1 cells with stable CHIP overexpression or knockout. These cells were subsequently assessed for proliferation, colony formation, and tumorigenic capacity in subcutaneous xenograft models. Lactate production, LDH activity, ATP levels, and histone lactylation were measured in CRC cells with altered CHIP expression. Mechanistically, Western blot, cycloheximide chase, immunoprecipitation, pull-down, and ubiquitination assays were conducted to dissect the underlying molecular events. Rescue experiments were finally performed both in vivo and in vitro to validate the functional relevance of this regulatory axis in CRC.

**Results:**

CHIP overexpression suppressed glycolysis and markedly inhibited CRC proliferation and tumorigenesis in vivo and in vitro. Mechanistically, CHIP directly bound LDHA via its TPR domain and promoted K48-linked polyubiquitination and proteasomal degradation of LDHA. This CHIP-mediated LDHA degradation reduced lactate production and subsequently decreased histone H3 lysine 18 lactylation (H3K18la), leading to transcriptional repression of cell cycle-related genes.

**Conclusion:**

This work identifies LDHA as a substrate of CHIP and demonstrates that CHIP acts as a critical negative regulator of the Warburg effect in CRC. The CHIP–LDHA axis thus couples metabolic with epigenetic reprogramming and represents a potential therapeutic target.

**Supplementary Information:**

The online version contains supplementary material available at https://doi.org/10.1007/s13402-026-01260-z.

## Introduction

Colorectal cancer (CRC) poses a persistent and growing global health threat, ranking as the third most common, and the second deadliest, cancer worldwide [1]. The gravity of this burden is evident across countries; in China, CRC was the second most diagnosed cancer and fourth leading cause of cancer death in 2022 [2], while projections for the US place it among the top three cancers in terms of both incidence and mortality in 2025 [3]. This high disease burden stands in stark contrast to the limited clinical efficacy of current strategies, which include surgery, chemotherapy, and targeted therapy [4]. This critical disconnect highlights the imperative to elucidate the underlying molecular drivers of CRC progression.

The E3 ubiquitin ligase CHIP (also known as STUB1), which is encoded on chromosome 16p13.3, is a central player in maintaining proteostasis. Comprising an N-terminal tetratricopeptide repeat (TPR) domain and a C-terminal U-box domain, CHIP facilitates the degradation of misfolded or client proteins via the ubiquitin–proteasome system [5]. In CRC, CHIP expression is frequently downregulated, and low CHIP expression is correlated with poor prognosis; thus, CHIP is a tumor suppressor and an independent prognostic factor [6]. Mechanistically, CHIP ubiquitinates and promotes the degradation of numerous oncogenic substrates, including Gal1 [6], GPX4 [7], and PKM2 [8], in CRC. Despite these advances, its full repertoire of substrates, particularly those involved in metabolic reprogramming, remains incompletely characterized.

Lactate dehydrogenase A (LDHA) catalyzes the conversion of pyruvate to lactate, a pivotal step in glycolysis. It is frequently overexpressed in cancers, fueling the Warburg effect to support tumor growth [9]. LDHA expression is tightly regulated at multiple levels. Transcriptionally, it is activated by HIF-1α [10], c-Myc [11], HOXC4 [12], ZBTB7B [13], and other factors. Post-transcriptionally, its mRNA stability is modulated by various microRNAs [14, 15] and m6A modifications [16, 17]. At the protein level, LDHA is subject to diverse post-translational modifications, including phosphorylation [18, 19], acetylation [20], succinylation [21], palmitoylation [22], and ubiquitination [23–25]. Several E3 ubiquitin ligases, such as TRIM3 [25], Fbw7 [23], and the CTLH complex [24], have been reported to regulate LDHA stability through ubiquitination. This highlights the complexity of LDHA protein homeostasis and suggests that different E3 ligases may fine-tune its abundance in a context-dependent manner. Given the central role of LDHA in cancer metabolism, comprehensive identification of its regulatory E3 ubiquitin ligases remains essential.

Although both CHIP and LDHA have been independently implicated in CRC, a direct functional link between them, especially one that influences tumor metabolism, has not been established. Here, we demonstrate that CHIP acts as a previously uncharacterized E3 ubiquitin ligase for LDHA, directly targeting it for ubiquitination and degradation. This mechanism provides a molecular basis for how ubiquitin-dependent proteolysis restrains glycolysis in CRC. Moreover, we extend these findings beyond metabolism by revealing that the CHIP–LDHA axis attenuates histone H3 lactylation, thereby introducing a novel epigenetic pathway through which CHIP influences colorectal tumorigenesis. These findings provide foundational insights into the molecular circuitry governing metabolic reprogramming and tumor development in colorectal cancer.

## Materials and methods

### Plasmids and antibodies

The Flag-LDHA plasmid was purchased from Beijing ABclonal Biotechnology. Myc-LDHA and shLDHA plasmids were constructed in this study. The primers for Myc-LDHA were as follows: forward, ggaattcATGGAGCAGAAACTCATCTCTGAAGAGGATCTGGCCACCatggcaactctaaaggatcagc; reverse, ggttaacTTAAAATTGCAGCTCCTTTTGGATCC. The shLDHA sequence was AACGGAGAAAGCCGTCTTAATTCCACACCAATTAAGACGGCTTTCTCCTTTTTTC. All other plasmids were previously constructed and maintained in our laboratory.

The anti-CHIP antiserum was generated by immunizing rabbits with purified GST-CHIP (143–303). Antibodies against β-actin, β-tubulin, and LDHA were purchased from Cell Signaling Technology (USA). Antibodies against Flag (A-7), HA, Myc (9E10), and GFP (FL) were obtained from Santa Cruz Biotechnology (USA). HRP-conjugated secondary antibodies were obtained from Beijing Zhongshan Golden Bridge Biotechnology.

### Cell culture and tumor tissues

HEK293T and DLD1 cells were maintained in our laboratory, and SW480 cells were kindly provided by Professor Zhai Yonggong’s laboratory. HEK293T and SW480 cells were cultured in DMEM (Macgene, China) supplemented with 10% fetal bovine serum (FBS) (Biological Industries, Israel) and 1% penicillin–streptomycin (Macgene, China). DLD1 cells were cultured in RPMI-1640 medium (Macgene, China) supplemented with the same supplements. All the cell lines were maintained at 37 °C in a humidified incubator with 5% CO_2_.

Human colorectal cancer tissue samples and matched adjacent nontumor tissues were obtained from the PLA General Hospital with appropriate ethical approval. The acquisition of these samples was approved by the institutional review boards/ethics committees for human research at the respective hospitals. All tissue samples, except for those with staining failures, were used for analysis.

### Generation of stable cell lines

SW480 and DLD1 cells were seeded in 6-well plates. When the cell density reached approximately 50%, the culture medium was replaced with 1 mL of fresh complete medium containing the respective lentivirus (HA-CHIP overexpression, CHIP knockout, or corresponding control viruses) and 1 mL of medium containing 2× polybrene (MCE^®^, China). After 24 h of incubation at 37 °C with 5% CO_2_, the virus-containing medium was aspirated and replaced with fresh complete medium. At 72 h post-infection, the medium was replaced with selection medium containing puromycin (Solarbio^®^, China). The stably transfected cell pools were then expanded for subsequent experiments.

### Cell proliferation assay

Cells from the experimental and control groups were digested, resuspended, counted, and seeded into 96-well plates. Each cell type was plated in at least three replicate wells. After cell attachment, the plates were transferred to the IncuCyte™ Zoom long-term cell dynamic imaging system. The system was programmed to automatically capture images at regular intervals under conditions of 37 °C and 5% CO_2_. Cell confluence was quantified automatically by IncuCyte software. The data were extracted and plotted with time on the horizontal axis and cell confluence on the vertical axis.

### Cell colony formation assay

Cells from the experimental and control groups were digested, resuspended, and counted. A defined number of cells (500 to 1000 cells per well) were seeded into 6-well culture plates, with three replicate wells for each condition. The plates were then incubated at 37 °C with 5% CO_2_ for 1–2 weeks, after which the medium was refreshed every two days. Once visible colonies had formed, the culture medium was aspirated. The cells were gently washed with PBS (Gibco™, USA) and fixed with 4% paraformaldehyde (Servicebio, China) for 20 min. The cells were subsequently stained with 0.2% crystal violet (MCE^®^, China), photographed, and counted.

### Tumor xenograft

Female BALB/c nude mice (4 weeks old) were housed under specific pathogen-free (SPF) conditions. Cell suspensions were prepared by resuspending SW480/HA-CHIP, SW480/CHIP KO, or their respective control cells in sterile PBS. A volume of 0.1 mL containing 1 × 10^7^ cells was subcutaneously injected into the bilateral axillary regions of each mouse. Tumor size was measured with a caliper every two days, and tumor volume was calculated using the following formula: volume (mm^3^) = length × width × height. At the experimental endpoint, the mice were euthanized, and the tumors were excised, weighed, and processed for further analysis (e.g., formalin fixation for IHC or snap freezing for protein extraction). All animals per group were included, no outlier exclusion criteria were applied, and the results are presented as mean ± SD to reflect the full range of variability. All animal experiments were approved by the Institutional Animal Care and Use Committee (IACUC) of the College of Beijing Normal University and were conducted in a double-blind manner.

### Metabolic assays (lactate, LDH, ATP)

Lactate content, lactate dehydrogenase (LDH) activity, and ATP levels in cell culture supernatants or lysates were measured using commercial assay kits (Nanjing Jiancheng Bioengineering Institute) strictly according to the manufacturer’s protocols. Briefly, for lactate measurement, the cell supernatant was collected and reacted with the provided working solution, and the absorbance was measured at 530 nm. To measure LDH activity, the cell lysates were incubated with the substrate mixture, and the reduction in the concentration of NAD⁺ was monitored at 450 nm. With respect to ATP content, the reaction of ATP and creatine to phosphocreatine, which is catalyzed by creatine kinase, was detected using the phosphomolybdate colorimetric method. All measurements were normalized to the total protein concentration or cell number.

### Reverse transcription-quantitative PCR (RT-qPCR)

Total RNA was extracted using TRIzol reagent (Ambion, USA) and reverse transcribed using the StarScript II RT Kit (GenStar, China). Quantitative PCR was performed using a QuantStudio™ 6 Flex instrument with the reverse transcription product as the template and specific primers for amplification. The primers for RRAS2 were as follows: forward, TAAAGGATCGTGATGAGTTCCCA; reverse, AATTTCCTGATAACCCGGACAAG. The primers for ASPM were as follows: forward, TGCAGTCCTCAGCGCAGTCT; reverse, TGGCCCCAACTTCCATCAT. The primers for TTK were as follows: forward, TGGCCAACCTGCCTGTTT; reverse, AATGCATTCATTTGCTGAAGAAGA. Relative gene expression was calculated using the 2^−ΔΔCt^ method.

### Western blotting

Total protein was extracted from cells or ground tumor tissues using RIPA lysis buffer (Beyotime, China) supplemented with protease and phosphatase inhibitors. Equal amounts of protein were separated by SDS–PAGE on 10% gels and subsequently transferred onto nitrocellulose membranes. The membranes were blocked with 10% nonfat milk (Yili, China) in TBST for 1 h at 37 °C and then incubated with primary antibodies diluted in blocking buffer overnight at 4 °C. After being washed with TBST, the membranes were incubated with HRP-conjugated secondary antibodies for 1 h at 37 °C. The protein bands were visualized using an enhanced chemiluminescence (ECL) substrate kit (Meilun, China) and imaged with a chemiluminescence imaging system.

### Protein degradation assay

For the protein degradation assay, HEK293T cells were transfected into 12-well plates. At 24 h post-transfection, the culture medium was replaced with fresh medium containing either the proteasome inhibitor MG132 (final concentration 10–20 µM, MCE^®^, China) or an equal volume of DMSO (MCE^®^, China) as a vehicle control. The cells were harvested 6 h later and analyzed by Western blotting.

### Cycloheximide (CHX) chase assays

For endogenous chase CHX assays, DLD1 cells stably expressing HA-CHIP and empty-vector control cells were treated with 50 µg/mL CHX (MCE^®^, China) to halt new protein synthesis. Cells were harvested at the indicated time points (e.g., 0, 4, 8, and 12 h) for Western blot analysis.

### Coimmunoprecipitation (Co-IP) assay

HEK293T cells transfected with the designated plasmids were lysed on ice for 30 min in IP lysis buffer (50 mM Tris-Cl (pH 7.6), 150 mM NaCl, 1 mM EDTA, 0.5% NP-40, and 10% glycerol) (MCE^®^, China) supplemented with Cocktail (cOmplete™, Germany). Whole-cell lysates were clarified by centrifugation. The supernatant was incubated with 2 µg of the specified antibody and 30 µL of Protein G-agarose beads (Santa Cruz Biotechnology, USA) at 4 °C for 8 h with gentle rotation. The beads were then washed three times with ice-cold lysis buffer. Bound proteins were eluted by boiling in 2× SDS–PAGE loading buffer and analyzed by Western blotting.

### GST pull-down assay

GST and GST-tagged CHIP proteins (full-length and truncated proteins) were expressed in *E. coli* and purified using Glutathione Sepharose 4B beads. HEK293T cells transfected with the Flag-LDHA plasmid were lysed. The cell lysates were then incubated with the GST or GST-fusion protein-bound beads at 4 °C for 8 h. After extensive washing with lysis buffer, the proteins bound to the beads were eluted with SDS–PAGE loading buffer and subjected to Western blot analysis using an anti-Flag antibody.

### In cellulo ubiquitination

HEK293T cells were transfected with the specified expression plasmids. At 24 h post-transfection, the cells were treated with 25 µM MG132 for 6 h to stabilize ubiquitinated proteins. The cells were then harvested and lysed in buffer A (containing 6 M guanidine hydrochloride (Sigma, USA), 0.1 M sodium phosphate dibasic/monobasic (Sigma, USA), and 10 mM imidazole (pH 8.0), (Sigma, USA)). The cells were then subjected to ultrasonic treatment. The whole-cell lysates were incubated with 50 µL of equilibrated (50%) Ni–NTA agarose at room temperature for 3 h. The agarose beads were subsequently washed twice with buffer A, followed by two additional washes with a mixture of buffer A and buffer T1 (in a 1:3 ratio), and finally washed once with buffer T1 (containing 25 mM Tris-Cl and 20 mM imidazole; pH 6.8). The precipitated proteins were eluted with 2 volumes of SDS–PAGE loading buffer (containing 250 mM imidazole) and subjected to SDS–PAGE electrophoresis, followed by Western blot analysis.

### Immunohistochemistry

Formalin-fixed, paraffin-embedded tissue sections were deparaffinized and rehydrated. Antigen retrieval was performed by heating the sections in 0.01 M citrate buffer (pH 6.0) (Beijing Zhong Shan-Golden Bridge Biological Technology, China). Endogenous peroxidase activity was quenched with 3% H_2_O_2_ (Beijing Zhong Shan-Golden Bridge Biological Technology, China). The sections were then blocked with normal goat serum and incubated with the specified primary antibodies at 4 °C overnight. After being washed, the sections were incubated with a biotinylated secondary antibody (Beijing Zhong Shan-Golden Bridge Biological Technology, China). Color development was performed using a DAB substrate kit (Beijing Zhong Shan Golden Bridge Biological Technology, China). The sections were counterstained with hematoxylin, dehydrated, and mounted.

IHC scoring was performed by a pathologist who was blinded to the sample groups. Staining intensity was scored as 0 (negative), 1 (weak), 2 (moderate), or 3 (strong). The percentage of positive cells was scored as 0 (0%), 1 (1–24%), 2 (25–49%), 3 (50–74%), or 4 (75–100%). The final IHC score (range 0–12) for each sample was calculated by multiplying the intensity score by the percentage score.

### Data analysis and statistics

All quantitative data are presented as the mean ± standard deviation (SD) from at least three independent experiments. Data analysis and graph plotting were performed using GraphPad Prism software. Statistical significance between two groups was determined using an unpaired, two-tailed Student’s t test. For multiple group comparisons, one-way or two-way ANOVA with an appropriate post hoc test was used. A p value of less than 0.05 was considered to indicate statistical significance (*p < 0.05; **p < 0.01; ***p < 0.001; ****p < 0.0001).

## Results

### CHIP inhibits colorectal cancer cell proliferation and tumor growth

**Fig. 1:**
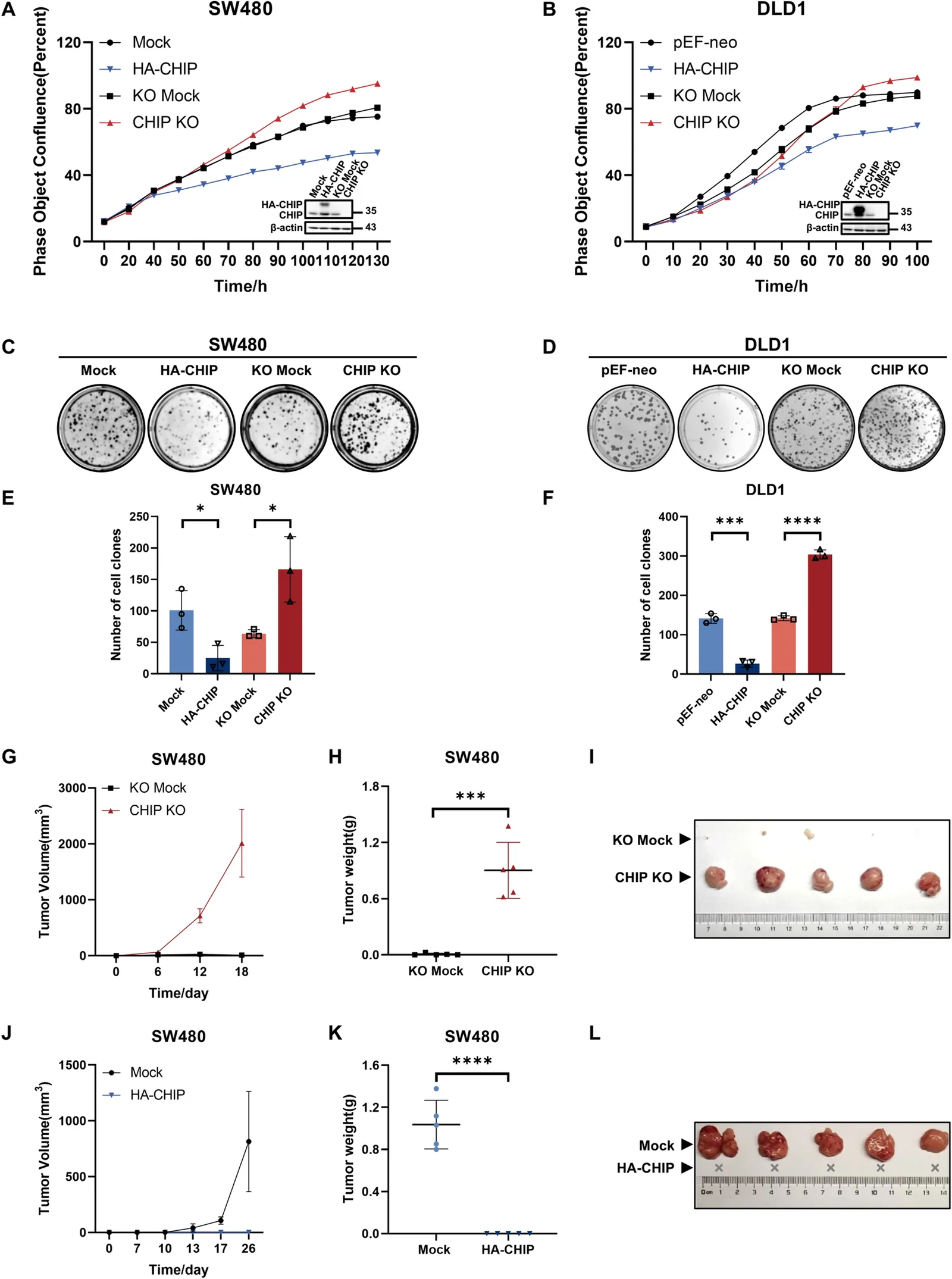
CHIP suppresses colorectal cancer cell growth in vivo and in vitro. (**A**) Assay for cell proliferation in SW480 cells stably overexpressing HA-CHIP or with CHIP knockout (KO). CHIP protein levels were detected by Western blotting, with β-actin used as a loading control. (**B**) Assay for cell proliferation in DLD1 cells stably overexpressing HA-CHIP or with CHIP knockout (KO). CHIP protein levels were detected by Western blotting, with β-actin used as a loading control. (**C-F)** Colony formation assays of SW480 (**C, E**) and DLD1 (**D, F**) cells with altered CHIP expression. Representative images (**C, D**) and quantitative analysis of colony numbers (**E, F**) are shown. (**G-I)** CHIP knockout promotes tumor growth in vivo. Nude mice were subcutaneously injected with SW480/CHIP KO cells (right flank) or control cells (left flank). Tumor volume (**G**), tumor weight (**H**), and representative images of excised tumors (**I**) are presented. (**J-L)** CHIP overexpression inhibits xenograft tumor growth. Nude mice were injected with 1 × 10^7^ SW480/HA-CHIP cells on the right side and control cells on the left side. Tumor size (**J**) and weight (**K**) are shown, with bar graphs representing the mean ± SD (*n* = 5). Representative tumor images are shown below (**L**). Data are presented as the mean ± SD (*n* = 5). Data in **A, B, E**, and **F** are presented as the mean ± SD for three independent experiments. Data in **A** (*p* < 0.0001), and **B** (*p* < 0.0001), **G** (*p* < 0.0001), and **J** (*p* < 0.0001) were analyzed by two‑way repeated‑measures ANOVA with Geisser‑Greenhouse correction. p‑values were calculated using two‑tailed t‑tests for the data in **E, F, H** and **K**.*p < 0.05; ***p < 0.001; ****p < 0.0001

To investigate the role of CHIP in CRC growth, we established stable CHIP-overexpressing and CHIP-knockout (KO) cell lines in SW480 and DLD1 human CRC cells (DLD1/HA-CHIP and DLD1/pEF-Neo, DLD1/CHIP KO and DLD1/KO Mock, SW480/HA-CHIP and SW480/Mock, SW480/CHIP KO and SW480/KO Mock). Western blot analysis confirmed successful CHIP overexpression and knockout (Fig. 1A and B). Using an Incucyte™ Zoom long-term cell dynamic imaging system, we found that CHIP overexpression significantly impeded cell proliferation, whereas CHIP KO accelerated proliferation in both cell lines (Fig. 1A and B). Consistent with these findings, colony formation assays revealed that CHIP overexpression decreased, whereas CHIP KO increased, colony number and size (Fig. 1C-F). These findings indicate that CHIP inhibits the proliferation and colony formation of colorectal cancer cells in vitro.

Furthermore, in vivo subcutaneous xenograft models demonstrated that compared with control tumors, tumors derived from CHIP-overexpressing SW480 cells grew more slowly, were smaller and weighed less (Fig. 1G-I). Conversely, CHIP KO promoted tumor growth (Fig. 1J-L). Collectively, these data indicate that CHIP acts as a potent suppressor of CRC growth.

### CHIP negatively regulates cellular glycolysis and histone H3 lactylation

**Fig. 2:**
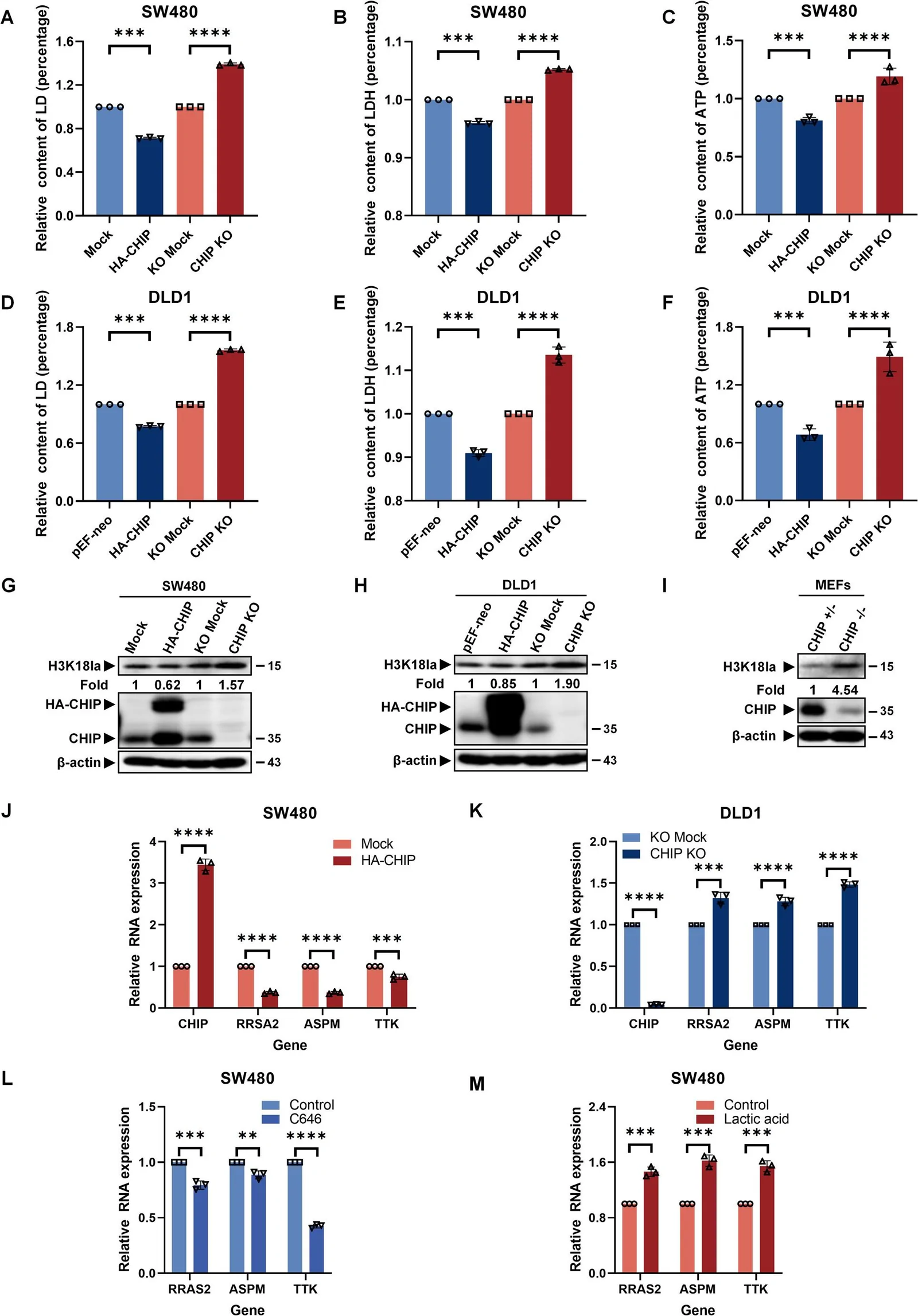
CHIP is a negative regulator of energy metabolism and histone H3 lactylation. (**A**) Measurement of lactate production (LD) in SW480 cells with modulated CHIP expression. (**B**) Measurement of lactate dehydrogenase (LDH) activity in SW480 cells with modulated CHIP expression. (**C**) Measurement of intracellular ATP levels in SW480 cells with modulated CHIP expression. (**D**) Measurement of lactate production (LD) in DLD1 cells with modulated CHIP expression. (**E**) Measurement of lactate dehydrogenase (LDH) activity in DLD1 cells with modulated CHIP expression. (**F**) Measurement of intracellular ATP levels in DLD1 cells with modulated CHIP expression. (**G**) Western blot analysis of H3K18la levels in SW480/HA‑CHIP and SW480/CHIP KO cells with respective controls. (**H**) Western blot analysis of H3K18la levels in DLD1/HA‑CHIP and DLD1/CHIP KO cells with respective controls. (**I**) Western blot analysis of H3K18la levels in heterozygous (CHIP+/−) and knockout (CHIP−/−) mouse embryonic fibroblasts (MEFs). (**J**) RT‑qPCR analysis of RRAS2, ASPM, and TTK transcript levels in CHIP‑overexpressing SW480 cells. (**K**) RT-qPCR was performed to measure the transcript levels of RRAS2, ASPM, and TTK in CHIP-knockout DLD1 cells. (**L**) RT‑qPCR analysis of RRAS2, ASPM, and TTK transcript levels in SW480 cells treated with the H3K18la inhibitor C646 (20 μM). (**M**) RT‑qPCR analysis of RRAS2, ASPM, and TTK transcript levels in SW480 cells treated with lactate (20 mM). Data in **A**–**F** and **J**–**M** are presented as the mean ± SD for three independent experiments. p‑values were calculated using two‑tailed t‑tests for the data in **A**–**F** and **J**–**M**. *p<0.05; **p<0.01; ***p<0.001; ****p<0.0001

We previously demonstrated that CHIP mediates the ubiquitination and degradation of the glycolytic enzyme PKM2 [26], thereby reducing glycolytic flux; In the same SILAD-based proteomic analysis, additional glycolytic enzymes targeted by CHIP were identified, including LDHA. Given CHIP’s specific targeting of these key glycolytic enzymes, we next investigated whether CHIP influences cellular energy metabolism in CRC. We measured key glycolytic parameters in CHIP-modulated SW480 and DLD1 cells. Lactate production (LD), lactate dehydrogenase (LDH) activity, and intracellular ATP levels significantly decreased upon CHIP overexpression but increased in CHIP KO cells (Fig. 2A-F).

Since lactate serves as a precursor for the novel epigenetic modification of histone lactylation, we examined the level of histone H3 lysine 18 lactylation (H3K18la). Notably, CHIP overexpression reduced H3K18la expression, whereas CHIP knockout increased it, in DLD1 cells (Fig. 2G and H). This effect was replicated in CHIP KO mouse embryonic fibroblasts (MEFs) (Fig. 2I). Studies have shown that RRAS2 [27], ASPM [28], and TTK [29] are regulated by H3K18la in head and neck squamous cell carcinoma, gastric cancer, and pancreatic ductal adenocarcinoma, respectively, and all three exert pro-tumorigenic effects. Therefore, we then examined the transcriptional levels of RRAS2, ASPM, and TTK. As expected, given the decreased H3K18la levels, CHIP significantly downregulated their expression (Fig. 2J and K).

To determine whether the transcriptional changes are causally dependent on lactylation rather than other consequences of CHIP modulation, we treated SW480 cells with the lactylation inhibitor or exogenous lactate. Treatment with C646, an H3K18la inhibitor, reduced the RNA levels of RRAS2, ASPM, and TTK, whereas lactate treatment increased their expression (Fig. 2L and M). These findings indicate that the transcriptional changes of these genes in CRC cells are specifically dependent on lactylation. Consistently, these results demonstrate that CHIP functions as a negative regulator of glycolysis and associated histone lactylation.

### CHIP interacts with and degrades LDHA

**Fig. 3:**
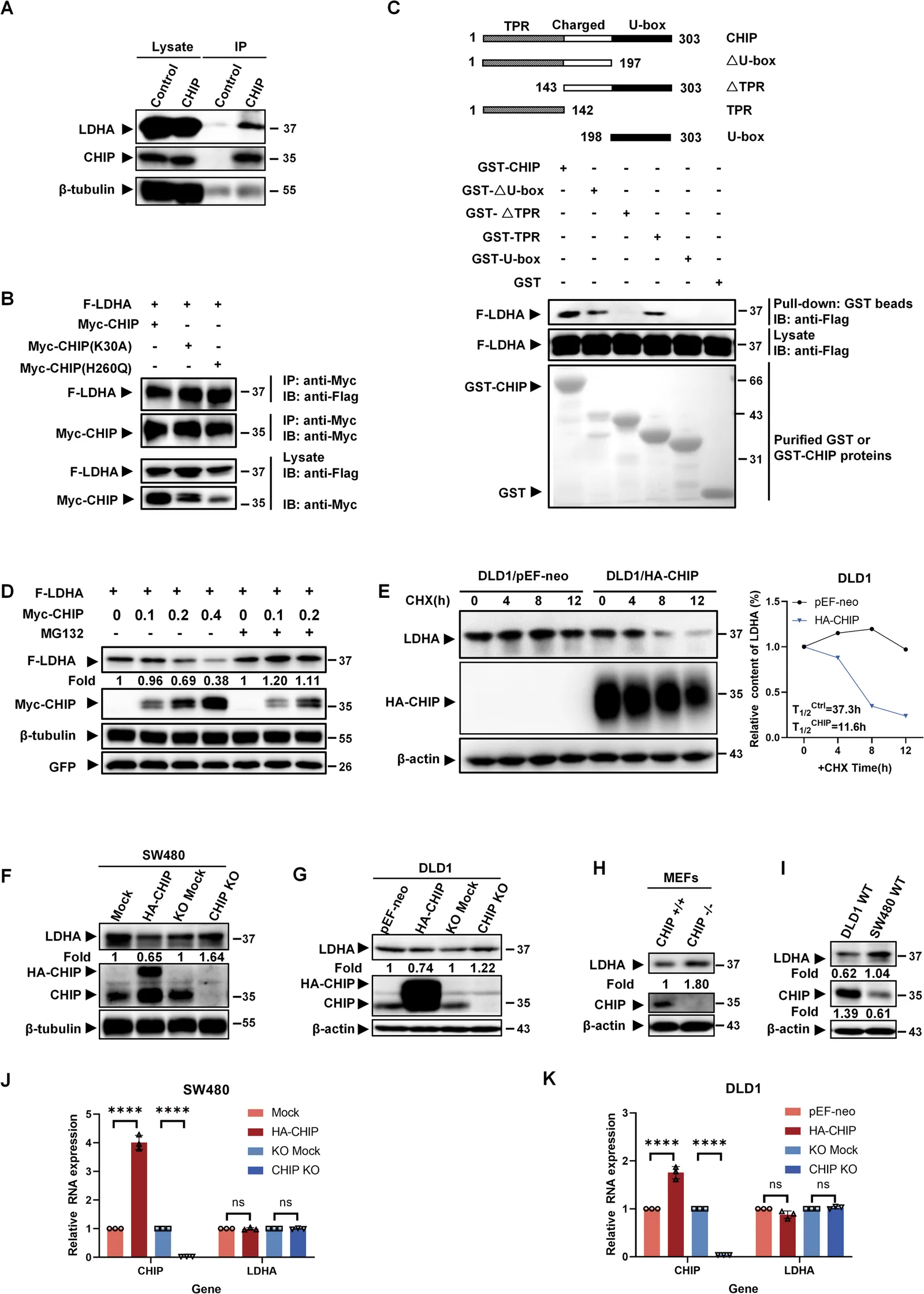
CHIP binds to and degrades LDHA. (**A**) Endogenous coimmunoprecipitation (Co-IP) in DLD1 cells. Cell lysates were immunoprecipitated with an anti-CHIP antibody or control IgG, followed by Western blot analysis for LDHA and CHIP. (**B**) Exogenous Co-IP in HEK293T cells. Cells were cotransfected with F-LDHA and Myc-CHIP (wild-type, K30A, or H260Q mutants). Lysates were immunoprecipitated with an anti-Myc antibody and blotted with the indicated antibodies. (**C**) GST pull-down assay. Purified GST or GST-CHIP (full-length and truncation mutant) fusion proteins were incubated with lysates from HEK293T cells expressing F-LDHA. The bound proteins were analyzed by Western blotting. (**D**) CHIP mediates LDHA degradation via the proteasome. HEK293T cells were cotransfected with fixed amounts of Flag-LDHA (F-LDHA) and GFP, along with increasing amounts of the Myc-CHIP plasmid. Cells were treated with or without MG132 (25 μM) for 6 h before being harvested. (**E**) Endogenous CHX chase assay. DLD1/HA-CHIP and control cells were treated with CHX (50 μg/mL) for the indicated times, and endogenous LDHA protein levels were analyzed. (**F**–**H**) Western blot analysis of LDHA expression in SW480 (**F**) and DLD1 (**G**) cells with modulated CHIP expression and in CHIP/WT and CHIP/KO MEFs (**H**). (**I**) Western blot analysis of LDHA and CHIP expression in DLD1 and SW480 cells. (**J**–**K**) RT-qPCR analysis of LDHA mRNA levels in SW480 (**J**) and DLD1 (**K**) cells following CHIP modulation. Data in **E**, **G**, and **K** are presented as the mean ± SD for three independent experiments. Data in **E** (p<0.0001) was analyzed by two‑way repeated‑measures ANOVA with Geisser‑Greenhouse correction. p‑values were calculated using two‑tailed t‑tests for the data in J and K. ns, not significant.; ****p<0.0001

To elucidate the mechanistic link between CHIP and glycolysis suppression, we focused on lactate dehydrogenase A (LDHA), a key enzyme that catalyzes the conversion of pyruvate to lactate. We first asked whether LDHA serves as a direct substrate of CHIP. Co-immunoprecipitation (Co-IP) assays confirmed that endogenous CHIP interacts with endogenous LDHA in CRC cells (Fig. 3A), an interaction that was recapitulated in exogenous Co-IP experiments in HEK293T cells (Fig. 3B). Notably, CHIP mutants deficient in chaperone binding (K30A) or E3 ligase activity (H260Q) retained LDHA binding (Fig. 3B), indicating a direct, chaperone-independent interaction. Domain mapping using GST pull-down assays with CHIP truncations revealed that the N-terminal TPR domain is both necessary and sufficient for direct binding to LDHA (Fig. 3C). Based on these observations, we hypothesized that CHIP, functioning as an E3 ubiquitin ligase, targets LDHA for degradation. Indeed, co-expression of Myc-CHIP led to a dose-dependent decrease in Flag-LDHA (F-LDHA) protein levels without affecting a co-transfected GFP control (Fig. 3D). This degradation was abolished by the proteasome inhibitor MG132 (Fig. 3D), demonstrating dependence on the ubiquitin–proteasome pathway. To directly assess LDHA stability, we performed cycloheximide (CHX) chase assays. Endogenous experiment showed that CHIP overexpression markedly shortened the half-life of LDHA protein (Fig. 3E). Accordingly, LDHA protein levels were strongly reduced upon CHIP overexpression, whereas CHIP knockout resulted in LDHA accumulation in both CRC cells and MEFs (Fig. 3F-H). Consistently, Western blot analysis of two CRC cell lines (DLD1 and SW480) revealed an inverse correlation between endogenous CHIP and LDHA protein levels (Fig. 3I). Meanwhile, we quantified LDHA mRNA levels in SW480 and DLD1 cells upon CHIP overexpression or knockout. Compared with respective controls, neither manipulation led to significant changes in LDHA mRNA levels, indicating that CHIP regulates LDHA expression at the protein level rather than by affecting its transcription (Fig. 3J and K). Collectively, these findings demonstrate that CHIP negatively regulates LDHA expression by promoting its proteasomal degradation.

### CHIP regulates LDHA stability via the ubiquitin-proteasome pathway

**Fig. 4:**
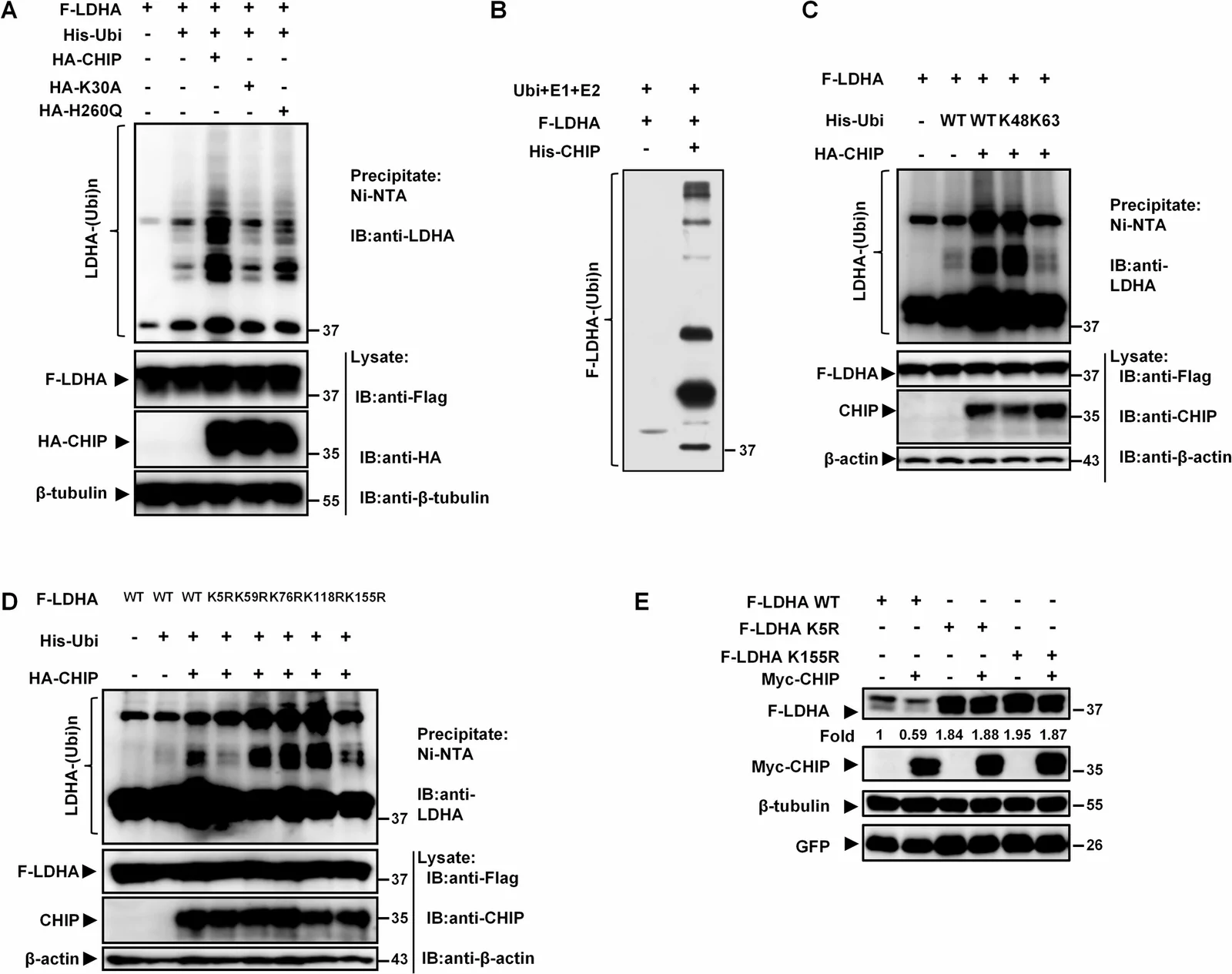
CHIP modulates LDHA stability through the ubiquitin-proteasome pathway. (**A**) In cellulo ubiquitination assay. HEK293T cells were cotransfected with F-LDHA, His-Ubiquitin, and HA-CHIP (wild-type, K30A, or H260Q mutants) as indicated and treated with MG132. His-ubiquitinated proteins were purified using Ni-NTA beads under denaturing conditions and analyzed by Western blotting with an anti-Flag antibody. (**B**) In vitro ubiquitination assay. Purified LDHA was incubated with E1, E2 (UbcH5a), HA-Ubiquitin, and purified GST-CHIP or GST control in reaction buffer. The reaction mixtures were analyzed by Western blotting with an anti-Flag antibody. (**C**) Analysis of ubiquitin linkage type. HEK293T cells were cotransfected with F-LDHA, HA-CHIP, and either wild-type His-Ubiquitin (His-Ubi-WT) or lysine-only mutants (His-Ubi-K48 or His-Ubi-K63). Ubiquitinated LDHA was analyzed as described in (**A**). (**D**) Identification of LDHA ubiquitination sites. HEK293T cells were cotransfected with HA-CHIP, His-Ubiquitin, and either wild-type F-LDHA (F-LDHA-WT) or the indicated lysine-to-arginine mutants. Ubiquitinated proteins were analyzed as described in (**A**). (**E**) CHIP mediates degradation of LDHA (WT, K5R, and K155R). HEK293T cells were cotransfected with fixed amounts of Flag‑LDHA (WT, K5R, or K155R), GFP, and Myc‑CHIP

To investigate the molecular mechanism by which CHIP regulates LDHA stability, we assessed whether CHIP directly ubiquitinates LDHA. *In cellulo* ubiquitination assays revealed that CHIP promoted the polyubiquitination of LDHA (Fig. 4A), which was confirmed in a cell-free in vitro ubiquitination system (Fig. 4B). Further analysis using ubiquitin mutants revealed that CHIP mediates K48-linked polyubiquitination of LDHA (Fig. 4C), a canonical signal for proteasomal degradation. Finally, we identified lysine residues at positions 5 and 155 (K5 and K155) on LDHA as the primary sites for CHIP‑mediated ubiquitination (Fig. 4D). Mutation of these residues (K5R and K155R) significantly reduced LDHA ubiquitination and, importantly, rendered the mutants resistant to degradation (Fig. 4E). In summary, CHIP is a bona fide E3 ubiquitin ligase that directly binds to LDHA and promotes its K48-linked ubiquitination at specific lysine residues.

### CHIP inhibits the proliferation of colorectal cancer cells by downregulating LDHA

**Fig. 5:**
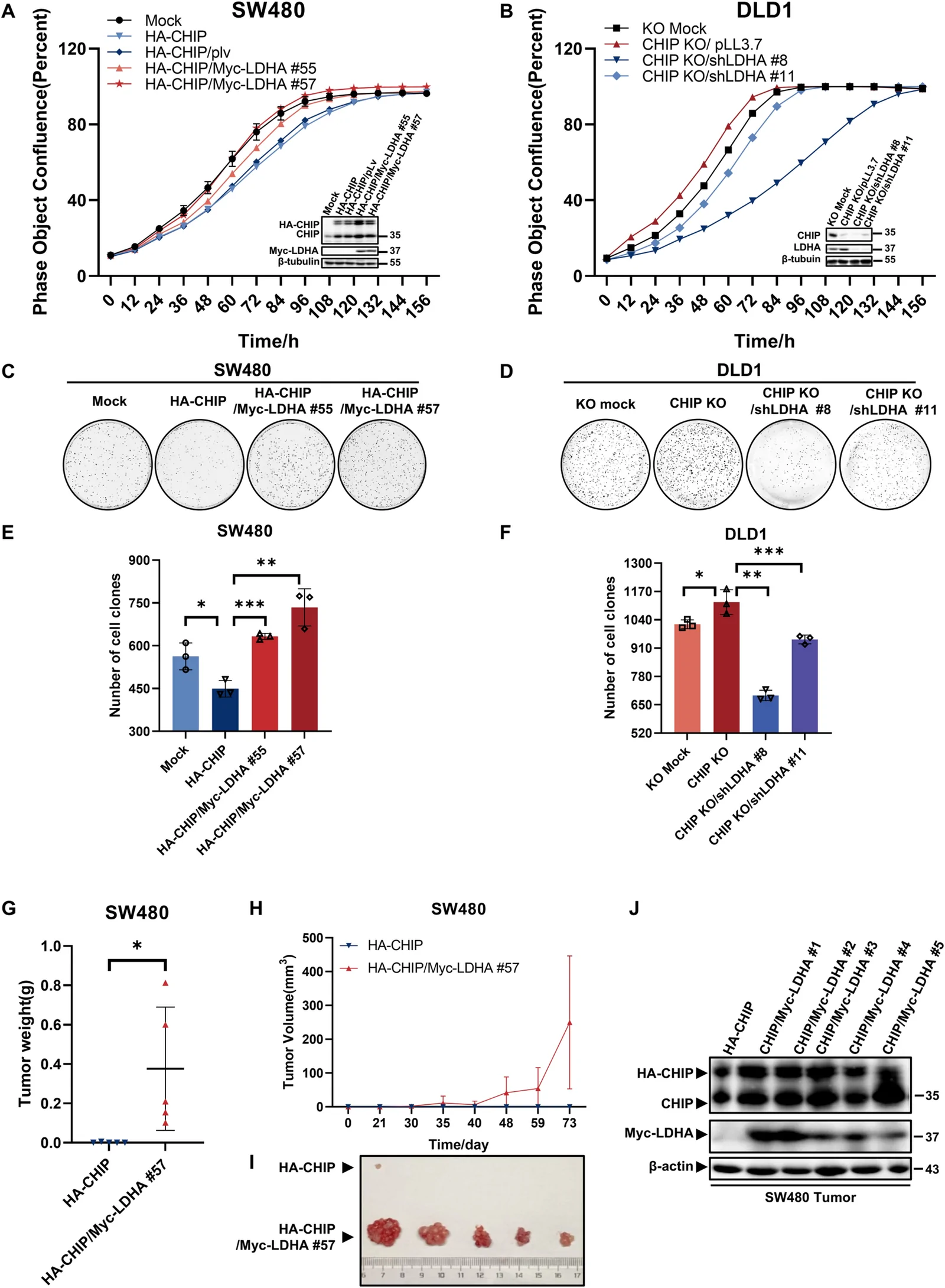
CHIP suppresses tumor cell proliferation and tumor growth accompanied by LDHA downregulation. (**A**) Proliferation curves of SW480/HA-CHIP cells following the re-expression of Myc-LDHA. Myc-LDHA and CHIP protein levels were detected by Western blotting, with β-actin used as a loading control. (**B**) Proliferation curves of DLD1/CHIP KO cells following LDHA knockdown. LDHA and CHIP protein levels were detected by Western blotting, with β-actin used as a loading control. (**C**-**F)** Colony formation assays corresponding to the conditions in **A** and **B**. Representative images (**C**, **D**) and quantitative analysis of colony numbers (**E**, **F**) are shown. (**G**-**J)** LDHA re-expression rescues the tumor-suppressive effect of CHIP in vivo. Nude mice were subcutaneously injected with SW480/HA-CHIP cells engineered to stably express Myc-LDHA or empty vector control. The tumor volume (**G**), tumor weight (**H**), representative images of excised tumors (**I**), and Western blot verification of protein expression in the stable cell lines (**J**) are presented. Data in **A**, **B**, **E**, and **F** are presented as the mean ± SD for three independent experiments. Data in **A** (*p* < 0.0001), **B** (*p* < 0.0001), and **H** (*p* < 0.0001) were analyzed by two‑way repeated‑measures ANOVA with Geisser‑Greenhouse correction. p‑values were calculated using two‑tailed t‑tests for the data in **E**, **F**, and **G**. All five animals per group were included, no outlier exclusion criteria were applied, and the results are presented as mean ± SD to reflect the full range of variability. *p < 0.05; **p < 0.01; ***p < 0.001; ****p < 0.0001

To determine whether the tumor-suppressive effect of CHIP is mediated through LDHA downregulation, we performed a series of rescue experiments. In SW480 cells, CHIP overexpression suppressed proliferation and colony formation, and these effects were significantly reversed by the reintroduction of LDHA (Fig. 5A and C, and 5E). Conversely, in DLD1 cells, the increased proliferation and colony formation resulting from CHIP knockout were effectively abolished by concomitant knockdown of LDHA (Fig. 5B and D, and 5F). We further validated these findings in vivo. The impaired tumor growth of CHIP-overexpressing SW480 cells was largely rescued by LDHA re-expression, resulting in increased tumor volume and weight (Fig. 5G-J). These genetic rescue experiments demonstrate that LDHA is a critical functional downstream target through which CHIP exerts its tumor-suppressive effects in CRC.

### CHIP negatively regulates energy metabolism and histone H3 lactylation by modulating LDHA

**Fig. 6:**
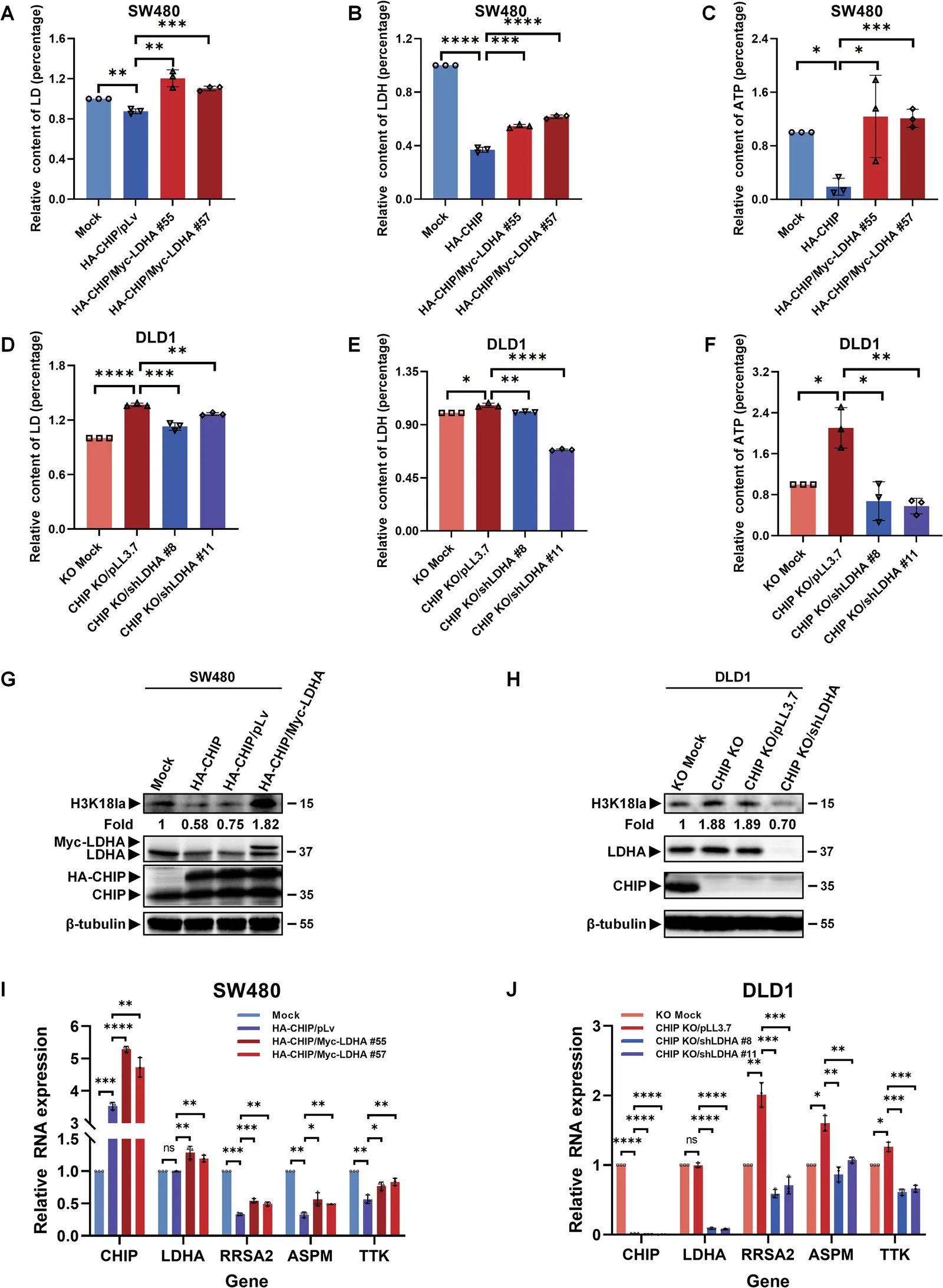
CHIP is a negative regulator of energy metabolism and histone H3 lactylation through its modulation of LDHA. (**A**–**C)** LD content, LDH activity, and intracellular ATP levels in CHIP‑overexpressing SW480 cells with or without additional LDHA re‑expression. (**D**–**F)** LD content, LDH activity, and intracellular ATP levels in CHIP‑knockout DLD1 cells with or without additional LDHA knockdown. (**G)** Western blot analysis of H3K18la levels in CHIP‑overexpressing SW480 cells with or without LDHA re‑expression (and respective controls). (**H)** Western blot analysis of H3K18la levels in CHIP‑knockout DLD1 cells with or without LDHA knockdown. (**I)** RT‑qPCR analysis of RRAS2, ASPM, and TTK transcript levels in CHIP‑overexpressing SW480 cells with or without LDHA re‑expression. (**J)** RT‑qPCR analysis of RRAS2, ASPM, and TTK transcript levels in CHIP‑knockout DLD1 cells with or without LDHA knockdown. Data in **A**–**F** and **I**–**J **are presented as the mean ± SD for three independent experiments. p‑values were calculated using two‑tailed t‑tests for the data in **A**–**F** and **I**–**J**. *p < 0.05; **p < 0.01; ***p < 0.001; ****p < 0.0001

To investigate whether CHIP influences cellular energy metabolism through its modulation of LDHA, we measured key glycolytic parameters in SW480 cells with CHIP overexpression and LDHA re‑expression, as well as in DLD1 cells with CHIP knockout and LDHA knockdown. Lactate content, LDH activity, and intracellular ATP levels significantly decreased upon CHIP overexpression but increased after LDHA re‑expression (Fig. 6A-C); conversely, these parameters increased upon CHIP knockout but decreased after LDHA knockdown (Fig. 6D-F). Meanwhile, we examined the level of histone H3 lysine 18 lactylation (H3K18la). Notably, CHIP overexpression reduced H3K18la levels, whereas LDHA re‑expression enhanced them (Fig. 6G); conversely, CHIP knockout increased H3K18la levels, while LDHA knockdown decreased them (Fig. 6H). We then examined the transcriptional levels of several reported H3K18la‑regulated genes implicated in cell proliferation—RRAS2, ASPM, and TTK. In line with the reduced H3K18la levels, CHIP significantly downregulated their expression, whereas re‑expressing LDHA increased H3K18la levels (Fig. 6I and J). Collectively, these results demonstrate that CHIP acts as a negative regulator of energy metabolism and histone H3 lactylation by modulating LDHA.

### The CHIP–LDHA axis is clinically relevant and underpins a tumor-suppressive mechanism in colorectal cancer

**Fig. 7:**
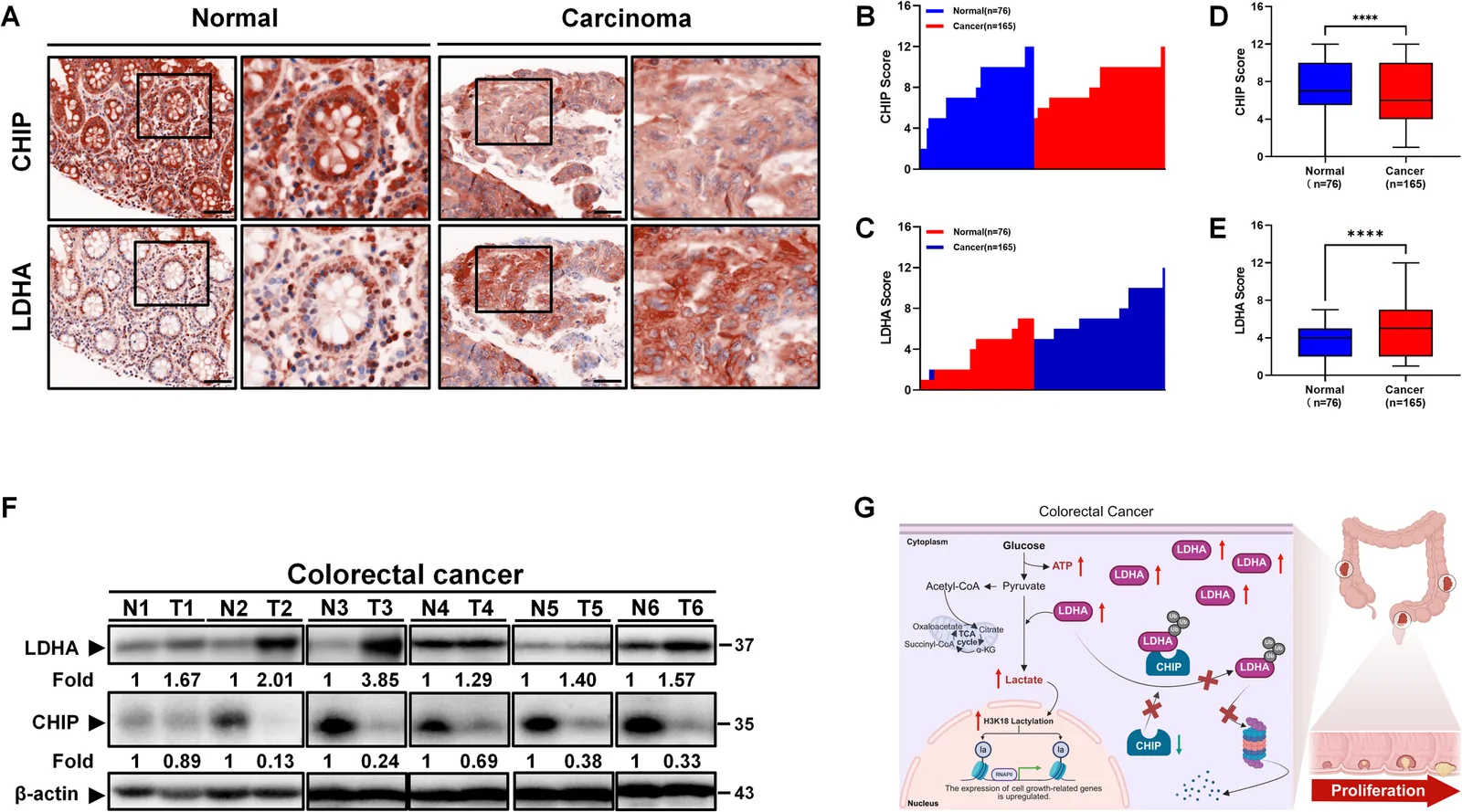
CHIP expression is inversely correlated with LDHA expression in human CRC specimens. (**A)** Representative immunohistochemistry (IHC) images showing CHIP and LDHA expression in normal colorectal epithelium and colorectal adenocarcinoma tissues. Scale bar, 50 μm. (**B**–**C**) Percentage of patients with high CHIP (**B**) or high LDHA (**C**) expression in normal (*n* = 76) and tumor (*n* = 165) tissues. (**D**–**E**) Quantitative analysis of IHC scores for CHIP (**D**) and LDHA (**E**) in unpaired normal and tumor tissues. (**F**) Western blot analysis of CHIP and LDHA protein levels in six pairs of human colorectal cancer (T) and matched adjacent nontumor (N) tissues. (**G**) A proposed model for the CHIP–LDHA tumor-suppressive axis in colorectal cancer. In colorectal cancer cells, the downregulation of CHIP leads to the accumulation of LDHA, enhancing glycolysis (the Warburg effect) and lactate production. Elevated lactate drives increased H3K18la levels, which in turn promotes the expression of genes involved in cell cycle progression, ultimately fueling tumor growth. p‑values were calculated using two‑tailed t‑tests for the data in **D** and **E**. ****p < 0.0001

To evaluate the clinical relevance of our findings, we analyzed CHIP and LDHA expression in a cohort of 284 human colorectal cancer specimens by immunohistochemistry (IHC). CHIP protein was highly expressed in normal epithelial cells but was significantly downregulated in adenocarcinoma cells. In stark contrast, LDHA expression was weak in normal tissues but strongly elevated in tumors (Fig. 7A). Statistical analysis confirmed that the frequency of high CHIP expression (Fig. 7B) and the average IHC score for CHIP (Fig. 7D) were significantly lower in cancer tissues, whereas the frequency of high LDHA expression (Fig. 7C) and its average IHC score (Fig. 7E) were markedly greater. Consistent with the IHC data, Western blot analysis of six paired clinical samples (tumor vs. adjacent normal tissue) consistently revealed an inverse relationship between CHIP and LDHA protein levels (Fig. 7F). These clinical data strongly support the pathological relevance of the CHIP–LDHA regulatory axis in human CRC.

Integrating these clinical observations with our mechanistic studies, we propose a comprehensive model for the tumor-suppressive role of CHIP in colorectal cancer (Fig. 7G). In CRC, the downregulation of CHIP impairs LDHA degradation, leading to its accumulation. The ensuing increase in glycolytic flux and lactate production elevates H3K18la levels, which transcriptionally activates genes that facilitate cell cycle progression, thereby accelerating colorectal cancer cell proliferation. Collectively, our findings from cellular, animal, and patient-derived samples establish the CHIP–LDHA axis as a critical and clinically relevant tumor-suppressive pathway in colorectal cancer.

## Discussion

Our study reveals a novel tumor-suppressive pathway in CRC in which the E3 ubiquitin ligase CHIP targets the glycolytic enzyme LDHA for ubiquitination and degradation. This CHIP–LDHA axis serves as a critical brake on glycolysis, histone lactylation, and, ultimately, tumor growth.

The ubiquitin–proteasome system employs a vast array of E3 ligases to maintain protein homeostasis. Currently, more than 600 types of E3 ubiquitin ligases encoded by the human genome are known [30]. CHIP, a U-box domain-containing E3 ligase, has been implicated in the degradation of diverse substrates, including several involved in cancer metabolism, such as eIF5A [31], p53 [32], p65 [33], CDK9 [34], FOXQ1 [35], PGK1 [36], and PKM2 [26]. Our current work adds LDHA to this growing list. Our findings reveal that CHIP orchestrates a multi-level regulation of lactate production by simultaneously targeting PKM2 and LDHA. While PKM2 determines the glycolytic flux and pyruvate availability, LDHA controls the final step of lactate generation. The dual regulation prevents potential compensatory lactate production when only one enzyme is inhibited, representing a more stringent mechanism to suppress the Warburg effect. Given the emerging roles of lactate in tumor immune evasion and metastasis, the CHIP-PKM2-LDHA axis may serve as a critical metabolic checkpoint. While other E3 ligases, including TRIM3 (a RING-domain protein) [25], Fbw7 [23], and the CTLH complex [24], have been reported to ubiquitinate LDHA, our identification of CHIP highlights the complex, multilayered regulation of LDHA protein stability. A network of E3 ligases likely collaborates to fine-tune LDHA levels in a tissue- and context-specific manner.

Our study provides mechanistic details that distinguish the CHIP–LDHA axis from previously reported pathways. First, we precisely mapped the interaction interface to the TPR domain of CHIP, which is distinct from the B-box and coiled-coil domains utilized by TRIM3 for LDHA binding [25]. Second, we unequivocally demonstrated that CHIP catalyzes the K48-linked polyubiquitination of LDHA, which targets it for proteasomal degradation. Most importantly, we identified specific lysine residues (K5 and K155) on LDHA that are modified by CHIP. This level of structural and mechanistic resolution is a meaningful step forward. Furthermore, recent studies highlight that LDHA undergoes complex post-translational crosstalk. Notably, acetylation at K5 has been shown to direct LDHA toward chaperone-mediated autophagy (CMA) for degradation [37], while our study demonstrates that CHIP-mediated ubiquitination also targets K5 for proteasomal degradation. This convergence suggests that K5 may act as a critical regulatory hub, potentially directing LDHA toward distinct degradation pathways (UPS vs. CMA) depending on the cellular context and competitive occupancy by acetylation or ubiquitination. Future studies dissecting whether these modifications compete or cooperate at the K5 residue will provide deeper insights into the precise metabolic control of LDHA.

A key finding of our study is a previously undescribed downstream epigenetic consequence of the CHIP–LDHA axis. We found that by degrading LDHA and reducing lactate production, CHIP attenuates histone H3 lysine 18 lactylation (H3K18la), a metabolite-sensitive modification that correlates with changes in gene expression. Crucially, we linked the reduction in H3K18la to the downregulation of key pro-proliferative genes, including RRAS2 [27], ASPM [28], and TTK [38], thereby establishing a functional connection from CHIP-mediated ubiquitination through metabolic rewiring and epigenetic alterations to the suppression of tumor cell proliferation. Direct binding of H3K18la to the promoters of these genes in CRC cells remains to be determined by future ChIP-qPCR or ChIP-seq studies. While H3K18la represents the core effector, the broader lactylation landscape and potential crosstalk with other metabolic modifications warrant systematic investigation in future studies. Another potential limitation of this study is that the metabolic characterization of the CHIP-LDHA axis was primarily established in microsatellite stable (MSS) CRC cell lines (SW480 and DLD1). Given the distinct molecular and metabolic features of microsatellite instability-high (MSI-H) CRC, future investigations using MSI-H models (e.g., HCT116) are required to fully evaluate the generalizability of CHIP-mediated lactylation regulation across different CRC subtypes.

The role of CHIP in cancer appears to be context dependent. A few studies have suggested that CHIP has an oncogenic function in specific settings [39, 40]. However, the predominant body of evidence, including findings from our laboratory and others, supports its role as a tumor suppressor in multiple cancer types [41–45], including CRC [33]. Our data consistently demonstrate that CHIP inhibits CRC cell growth in vivo and in vitro and that CHIP loss leads to LDHA accumulation and enhanced glycolytic flux. A potential limitation of this study is the lack of direct measurement of glucose consumption or extracellular acidification/oxygen consumption rates (e.g. via Seahorse). Nevertheless, our integrated analysis of lactate production, LDH activity, and ATP data consistently support a model in which CHIP negatively regulates glycolytic flux (Fig. 2C and F). Notably, the observed increase in ATP upon CHIP knockout which seems counterintuitive given the lower per-glucose ATP yield of glycolysis compared to OXPHOS—aligns well with the high-flux state of the Warburg effect [46]. In this state, cancer cells drastically upregulate glucose uptake (e.g., via GLUT1) [47], and exploit the significantly faster ATP generation rate of glycolysis [48]. Consequently, the sheer volume of glucose processed compensates for the low efficiency per molecule, resulting in a net increase in total ATP. Future studies using comprehensive metabolic flux analysis will further delineate the full metabolic consequences of the CHIP‑LDHA axis. The negative correlation between CHIP and LDHA protein levels in a large cohort of clinical CRC samples further reinforces the physiological relevance of our proposed axis.

In conclusion, our findings substantially expand the known functional repertoire of CHIP, suggesting that it plays a central role in negatively regulating aerobic glycolysis via LDHA degradation. The CHIP–LDHA axis represents a crucial metabolic and epigenetic checkpoint in CRC. Therapeutic strategies aimed at restoring CHIP function or mimicking its activity on LDHA could therefore open new avenues for intervention not only in CRC but also potentially in other Warburg-dependent malignancies.

## Conclusion

In colorectal cancer, CHIP deficiency promotes tumor progression by impairing LDHA degradation, leading to enhanced glycolytic flux and lactate production. The accumulated lactate elevates H3K18la levels, which is associated with increased expression of cell cycle regulators including RRAS2, ASPM, and TTK, thereby accelerating tumor growth. Collectively, our multi-level evidence from cellular, animal, and clinical samples establishes the CHIP–LDHA axis as a critical tumor-suppressive pathway and potential therapeutic target in colorectal cancer.

## Supplementary Information

Below is the link to the electronic supplementary material.

Supplementary Material 1

## Data Availability

No datasets were generated or analysed during the current study.

## Abbreviations

CHIP: Carboxyl terminus of Hsp70-interacting protein
STUB1: STIP1 homology and U-box containing protein 1
LDHA: Lactate dehydrogenase A
CRC: Colorectal cancer
H3K18la: Histone H3 lysine 18 lactylation
TPR: Tetratricopeptide repeat
Gal1: Galectin-1
GPX4: Glutathione peroxidase 4
PKM2: Pyruvate kinase M2
HIF-1α: Hypoxia-inducible factor 1-alpha
c-Myc: MYC proto-oncogene protein
HOXC4: Homeobox C4
ZBTB7B: Zinc finger and BTB domain-containing protein 7B
TRIM3: Tripartite motif-containing protein 3
Fbw7: F-box/WD repeat-containing protein 7
CTLH complex: C-terminal to LisH complex
KO: Knockout
LDH: Lactate dehydrogenase
MEFs: Mouse embryonic fibroblasts
RRAS2: RAS-related protein 2
ASPM: Abnormal spindle-like microcephaly-associated protein
TTK: TTK protein kinase
CHX: Cycloheximide
Co-IP: Coimmunoprecipitation
IHC: Immunohistochemistry
eIF5A: Eukaryotic translation initiation factor 5A
p53: Cellular tumor antigen p53
p65: Transcription factor p65
CDK9: Cyclin-dependent kinase 9
FOXQ1: Forkhead box protein Q1
pGK1: Phosphoglycerate kinase 1
IACUC: Institutional Animal Care and Use Committee
